# Monitoring the effects of iodine prophylaxis in the adult population of southern Italy with deficient and sufficient iodine intake levels: a cross-sectional, epidemiological study

**DOI:** 10.1017/S0007114516004499

**Published:** 2017-01-18

**Authors:** Daniela Bonofiglio, Stefania Catalano, Anna Perri, Marta Santoro, Lorenza Siciliano, Danilo Lofaro, Massimiliano Gallo, Stefania Marsico, Rosalinda Bruno, Cinzia Giordano, Ines Barone, Sebastiano Andò

**Affiliations:** 1 Department of Pharmacy, Health and Nutritional Sciences, University of Calabria, 87036 Arcavacata di Rende (CS), Italy; 2 Centro Sanitario, University of Calabria, 87036 Rende (CS), Italy; 3 Department of Nephrology, Dialysis and Transplantation, Kidney and Transplantation Research Center, Annunziata Hospital, 87100 Cosenza, Italy

**Keywords:** Iodine intake, Thyroid diseases, Thyroid antibodies, Goitre

## Abstract

I prophylaxis is the most effective strategy to eradicate I deficiency disorders, but it has been shown to affect the thyroid disease pattern. In this study, we assessed the frequency of thyroid disorders in an adult population living in two areas of southern Italy after implementing I prophylaxis. To this aim, a cross-sectional, population-based study including 489 subjects from an I-deficient rural and an I-sufficient urban area of southern Italy was conducted. Thyroid ultrasound was performed on all participants, and urine and blood samples were collected from each subject. The levels of thyroid-stimulating hormone (TSH), thyroglobulin (TgAb) and thyroperoxidase antibodies (TPOAb), urinary I excretion (UIE), and thyroid volume and echogenicity were evaluated. We found that the median UIE was higher in the urban than in the rural area (*P*=0·004), whereas the prevalence of subjects affected by goitre was higher in the rural compared with the urban area (*P*=0·003). Positive TgAb rather than TPOAb were more frequent in subjects from the urban area compared with the rural area (*P*=0·009). The hypoechoic pattern at thyroid ultrasound (HT-US) was similar between the two areas, but TgAb were significantly higher (*P*=0·01) in HT-US subjects from the urban area. The frequency of elevated TSH did not differ between the two screened populations, and no changes were found for TgAb positivity in subjects with high TSH in the urban compared with the rural area. Our findings support that the small risks of I supplementation are far outweighed by the substantial benefits of correcting I deficiency, although continued monitoring of populations is necessary.

Thyroid disorders such as goitre, nodules, autoimmune disease and thyroid dysfunction affect many people worldwide. The spectrum and prevalence of thyroid diseases depend on age, sex, ethnicity and environmental factors, especially on I intake^(^ ^1^ ^–^ ^3^ ^)^. As one of the major public health problems, I deficiency disorders have several consequences on human health, ranging from defective development of the central nervous system during fetal–neonatal life to goitre in adulthood^(^ ^4^ ^,^ ^5^ ^)^. I prophylaxis using iodised salt, recommended by the WHO and the Iodine Global Network, has been shown not only to exert an important role in eradicating I deficiency disorders but also to influence the thyroid disease pattern^(^ ^6^ ^)^. Indeed, several studies carried out on populations living in areas with different I intake have demonstrated that increase in I intake in I-deficient countries may determine hyperthyroidism^(^ ^7^ ^,^ ^8^ ^)^. On the other hand, a higher frequency of thyroid autoimmunity and hypothyroidism has been reported in I-sufficient than in I-deficient populations^(^ ^9^ ^,^ ^10^ ^)^. In line with these findings, longitudinal studies conducted in Denmark have also highlighted an increased incidence of thyroid autoantibodies^(^ ^11^ ^)^ and hypothyroidism^(^ ^12^ ^)^ after adopting I prophylaxis. In addition, more than adequate I intake has been associated with thyroid disorders such as goitre, hypothyroidism and autoimmune thyroiditis^(^ ^13^ ^–^ ^15^ ^)^. Recently, a reduction in the prevalence of goitre and thyroid autonomy in younger subjects along with a decreased frequency of non-autoimmune hyperthyroidism in older subjects has been reported in a small southern Italian village following a 15-year period of voluntary I prophylaxis. An increase in serum thyroid antibodies and hypothyroidism, particularly in its subclinical form, was observed in the same area^(^ ^16^ ^)^. The authors suggest that I induces thyroid autoimmunity by unmasking a cryptic epitope on thyroglobulin (TgAb)^(^ ^17^ ^)^. In addition, the use of GM animal models has provided evidence that I intake can precipitate spontaneous autoimmune thyroid disease by increasing the immunogenicity of TgAb^(^ ^18^ ^)^.

For more than two decades, we have carried out studies on the prevalence of goitre in the populations living in southern Italy either in areas of adequate I intake or in communities with severe-to-moderate I deficiency. The introduction of voluntary I supply programmes in I-deficient areas resulted in increased urinary I excretion (UIE) together with decreased goitre prevalence^(^ ^19^ ^,^ ^20^ ^)^. Despite the clear benefits of I prophylaxis, a continuous surveillance of I-induced adverse effects needs to be carefully recommended.

In the present study, we aimed to assess the frequency of thyroid disorders including goitre and nodules along with the levels of thyroid antibodies in the adult population of a small village previously investigated for I deficiency and in an I-sufficient town in the southern Italian region of Calabria.

## Methods

### Subjects

A total of 560 subjects (155 males; 405 females) were initially enrolled for this survey, which was carried out between July and November 2015. The subjects belonged to an adult population born and living in rural (274 subjects) and urban (286 subjects) areas in the southern Italian region of Calabria. The project was strongly supported by civil and health authorities, and informed written consent was obtained from all subjects. Ethical committees approved the protocol of the present study.

A questionnaire was completed including personal and family history of thyroid diseases. As we enrolled only working-age subjects in the urban area, seventy-one subjects aged <25 or >65 years from the rural area were excluded to match age between groups, thus resulting in 489 participants with 203 and 286 subjects from the rural and urban area, respectively. Sample size was calculated using the following formula derived from Cohen’s statistical power analysis:
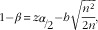
where *α* is the Type I error, *β* the Type II error, meaning 1−*β* is power, 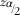 the two-sided *Z*-score for the Type I error and *h* the effect size calculated as 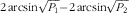. The calculated minimum sample size was 191 subjects for each group. Given the number of participants in the two areas and the difference in the prevalence of goitre, we had an effect size of 0·287, resulting in a study statistical power of 87·8 %.

In addition, thyroid-stimulating hormone (TSH) levels were determined in 447 samples after excluding forty-two subjects (thirteen and twenty-nine from the rural and urban area, respectively) for using thyroid medication (e.g. treatment with levothyroxine or antithyroid drugs). Alimentary habits and the use of iodised salt were also evaluated. In all, 131 subjects (64·5 %) from the rural area and 235 subjects (82·2 %) from the urban area declared using iodised salt routinely, whereas the coverage rate of iodised salt was approximately 65 % in the region of Calabria. Median I concentration in the iodised salt was 30 (interquartile range (IQR) 28–32) mg/kg.

Results were compared with data from a previous survey conducted in the same rural area in 2007^(^ ^23^ ^)^. At that time, 707 adults (185 males and 522 females) had been enrolled, and goitre prevalence was evaluated using the same sonographic criteria reported below. UIE was measured in 600 subjects using the methods described below.

#### Thyroid ultrasound

Thyroid volume (TV) was estimated using a real-time, ultrasound (Logic α100; General & Electrics Medical Systems), portable instrument, with a 10-MHz linear transducer. Thyroid ultrasound was performed by two expert physicians. The subjects were examined in the supine position, with the neck hyperextended. TV was calculated by using the formula of a rotation ellipsoid model: width×length×depth×0·52 for each lobe. Isthmus volume was not taken into account. Thyroid enlargement was defined as a TV >18 ml for women and >25 ml for men, which corresponds to the mean+3 sd in I-sufficient populations as previously reported^(^ ^21^ ^)^.

#### Laboratory evaluation

To evaluate the UIE, we collected morning spot urinary samples into tubes washed with de-ionised water and stored at −20°C until analysis. UIE was measured using a manual spectrophotometric method based on the Sandell–Kolthoff reaction, as described by Dunn *et al*.^(^ ^22^ ^)^. I concentration was expressed as µg/l, and the I-deficiency grade was defined according to the WHO’s median UIE level criteria^(^ ^23^ ^)^.

Serum TSH was determined using the ^125^I-hTSH IRMA system that provides direct quantitative *in vitro* determination of this human hormone (Institute of Isotopes Co.). TSH levels, expressed in µIU/ml, were measured using a Packard RIASTAR Gamma Counter (Packard Instrument Company). Imprecision was determined by analysing the two levels of commercial control materials (low and high). The levels of TSH in euthyroid controls ranged from 0·35 to 4·0 μIU/ml.

TgAb and thyroperoxidase antibodies (TPOAb) were measured by an automated immunoassay system (Zentech s.a.) using a Packard RIASTAR Gamma Counter. TgAb and TPOAb levels were negative when <30 and 60 U/ml, respectively.

#### Statistical analysis

Results of UIE are presented as medians and IQR. A non-parametric test (*χ* ^2^, Fisher exact test, Mann–Whitney *U* test) was used as appropriate and considered statistically significant when *P*<0·05. All the analyses were performed using R (version 3.2.3; The R Foundation for Statistical Computing).

## Results

**Fig. 1:**
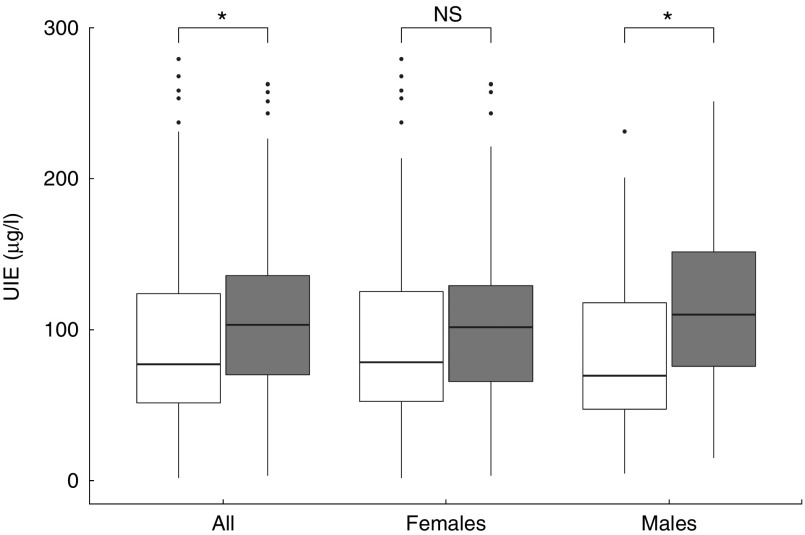
Box-whisker plot of urinary I excretion (UIE) measured in rural (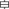) and urban (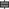) areas. Values are medians (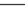) and interquartile ranges (IQR; 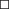, 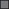) and data within 1·5× IQR of the 1st and 3rd quantile, respectively. Data beyond the end of the whiskers are outliers plotted as points. *P<0·05.

A total of 489 subjects were analysed in the present study. Subjects were recruited from an adult population born and living in a small village (rural area=203), previously investigated for I deficiency, and in an I-sufficient town (urban area=286) in the region of Calabria in southern Italy. The features of the population studied are reported in Table 1. The percentage of subjects that declared to routinely use iodised salt was 64·5 and 82·2 %, from the rural and the urban areas, respectively. The median UIE was 77·1 (IQR 51·2–124) µg/l in the rural area and 103·3 (IQR 70·2–136) µg/l in the urban area (*P*=0·004), thus confirming the heterogeneity from mild/moderate to sufficient in I supply among different areas of southern Italy. This difference was higher in males (median 69·6 (IQR 47·3–117·9) µg/l rural *v.* 110·1 (75·8–151·4) µg/l urban area, *P*=0·009) than in females (median 78·5 (IQR 52·5–125·3) µg/l rural *v.* 101·7 (IQR 65·7–129·3) µg/l urban area, *P*=0·07) (Fig. 1).

**Table 1:** Features and urinary iodine excretion (UIE) of the population studied living in an iodine-deficient (rural) and in an iodine-sufficient (urban) area of southern Italy (Mean values and standard deviations; medians and interquartile ranges (IQR))

	Rural area	Urban area
Sample size (*n*)	203	286
Sex (M:F)	42:161	96:190
Age (years)		
Mean	49·2	49·8
sd	10·6	9·1
UIE (μg/l)		
Median	77·1	103·3
IQR	51·2–124	70·2–136

**Fig. 2:**
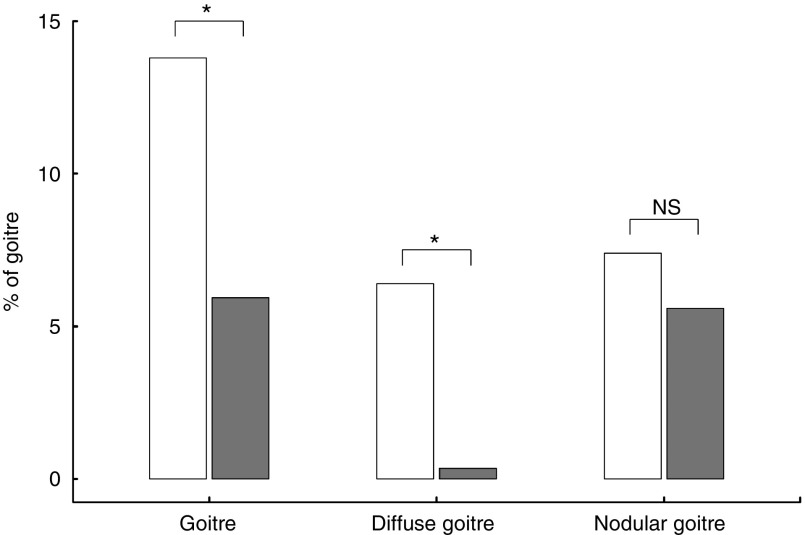
Frequency (%) of goitre, diffuse goitre and nodular goitre in subjects resident in rural (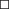) and urban (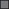) areas. *P<0·05.

**Fig. 3:**
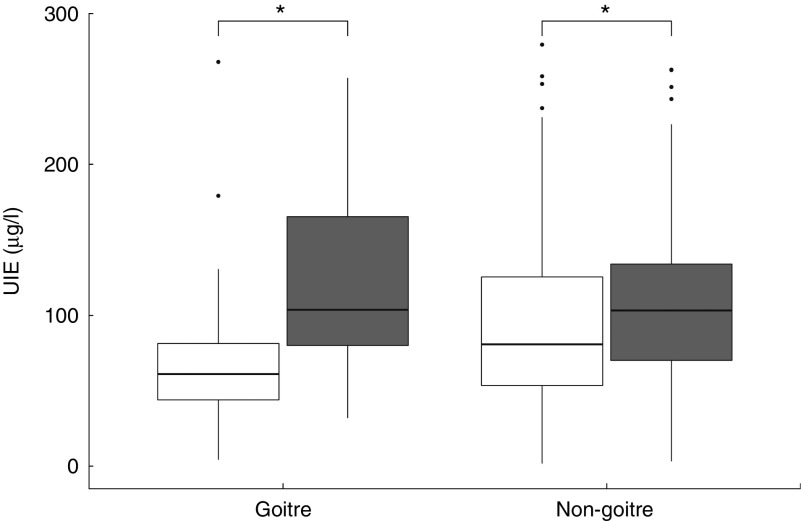
Box-whisker plot of urinary I excretion (UIE) measured in rural (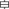) and urban (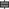) areas according to the presence of goitre. Values are medians (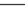) and interquartile ranges (IQR; 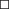, 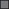) and data within 1·5× IQR of the 1st and 3rd quantile, respectively. Data beyond the end of the whiskers are outliers plotted as points. *P<0·05.

The prevalence of subjects affected by goitre was significantly higher in the rural than in the urban area (*P*=0·003) (Fig. 2), with a similar trend for both sexes (13·5 % rural *v.* 5 % urban area in females; 14 % rural *v.* 6·5 % urban area in males). When we analysed the cause of goitre, we found that the prevalence of diffuse goitre was higher in the rural than in the urban area (*P*<0·0001), whereas no significant difference was observed in nodular goitre between the two populations (Fig. 2). Difference in the UIE was more evident in subjects affected by goitre (median 61·1 (IQR 44·0–81·4) µg/l rural *v.* 103·7 (IQR 31·8–165·2) µg/l urban area, *P*=0·006) compared with unaffected subjects (median 80·8 (IQR 53·5–125·5) µg/l rural *v.* 103·3 (IQR 70·2–134) µg/l urban area, *P*=0·01) (Fig. 3).

We compared our results of UIE and goitre prevalence obtained from subjects of the rural area with data from the previous survey conducted in the same village in 2007^(^ ^20^ ^)^. UIE was numerically lower, but within the same range of the levels reported in 2007 (median 97 (IQR 62·5–132·2) µg/l rural area in 2007 *v*. 77·1 (IQR 51·2–124) µg/l rural area in 2015, *P*=0·069), whereas goitre prevalence had decreased (42·6 % rural area in 2007 *v*. 13·8 % rural area in 2015, *P*<0·0001).

Thyroid nodules were detected in 164 subjects (33·5 %), with a slight prevalent increase in the urban compared with the rural area (36·4 *v*. 29·6 %, *P*=0·104). The proportion of nodular disease in males was similar in both areas (35·7 *v.* 34·4 %, *P*=0·9), whereas it was significantly higher in females in the urban compared with those in the rural area (38·6 *v.* 28·5 %, *P*=0·03).

**Fig. 4:**
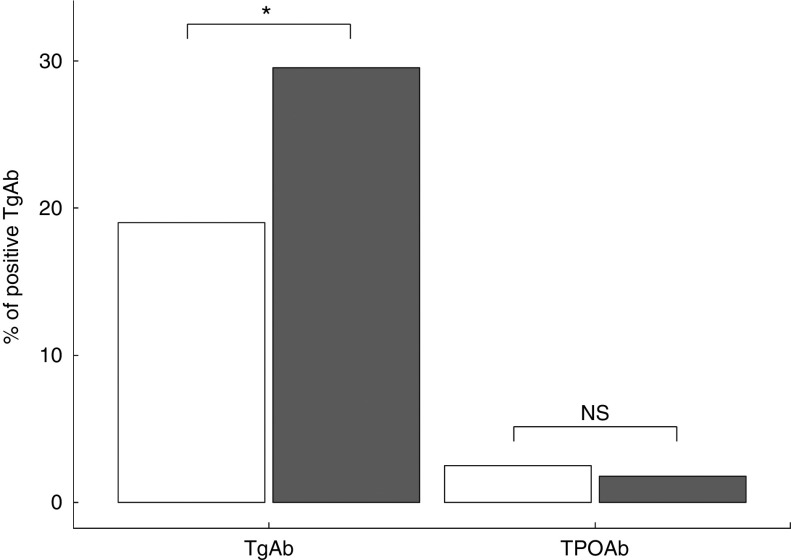
Bar plot representing frequency (%) of positive thyroglobulin (TgAb) and thyroperoxidase antibodies (TPOAb) in subjects resident in rural (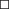) and urban (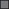) areas. *P<0·05.

**Fig. 5:**
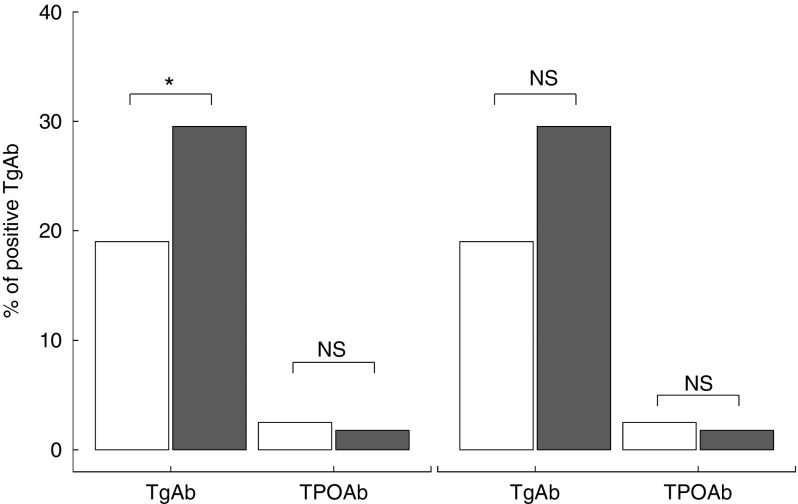
Frequency (%) of positive thyroglobulin (TgAb) and thyroperoxidase antibodies (TPOAb) in subjects with a hypoechoic pattern at thyroid ultrasound (HT-US) and normal hypoechoic pattern (non-HT-US) resident in rural (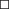) and urban (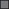) areas. *P<0·05.

Serum positivity of TgAb was significantly higher in subjects from the urban area (*P*=0·009), whereas the frequency of positive TPOAb (*P*=0·369) was similar between the screened populations (Fig. 4). Hypoechoic patterns at thyroid ultrasound (HT-US) did not differ between the two areas (24·7 % urban *v.* 19·3 % rural area, *P*=0·16). Serum levels of TgAb were higher only in HT-US subjects (Fig. 5) in the urban area compared with those in the rural area (*P*=0·01).

The median of TSH value differed significantly between the two investigated areas (median 1·3 µg/l rural *v.* 1·5 µg/l urban area, *P*=0·004). The frequency of elevated TSH (≥4 µUI/ml) was similar in the two screened areas (3·2 % rural *v.* 5·8 % urban area, *P*=0·2), whereas low TSH (≤0·35 µUI/ml) was found more frequently in subjects from the rural area (9·5 % rural *v.* 3·5 % urban area, *P*=0·01). Interestingly, no significant differences were found for TgAb positivity in the urban with respect to the rural area (9·8 *v.* 7·68 %, *P*=0·87) for subjects with TSH serum levels ≥4 μIU/ml.

## Discussion

The results of the present study confirm and extend our previous data^(^ ^19^ ^,^ ^20^ ^)^, indicating the benefits of the persistent iodoprophylaxis campaign in the I-deficient areas of the region of Calabria. Following recommendations from WHO in 1993, I prophylaxis is monitored by assessing both goitre prevalence and UI concentration^(^ ^24^ ^)^. We observed decreased goitre prevalence, mainly due to the reduction of diffuse goitre, in the adult population living in an urban area with a documented long-standing I intake with respect to that of a rural area previously investigated for I deficiency.

In our surveyed population, UI concentration, a reliable index of recent dietary I intake, indicated an adequate assumption in the urban area and a persistent I deficiency in the rural area. We carried out previous investigations in rural areas of the region of Calabria in the early 1990s, demonstrating a heterogeneity in I supply, ranging from sufficient to moderate with pockets of severe I deficiency. A 2-year programme of I supplementation resulted in an increased UIE together with a decreased goitre prevalence, suggesting the usefulness of an effective iodoprophylaxis in this region^(^ ^19^ ^)^. On the basis of these results, we strongly supported the use of iodised salt in the I-deficient areas, and this process was further reinforced in 2005 when I salt supplementation was introduced by law. Furthermore, results from a survey conducted in the same area in 2007 demonstrated that, in spite of the conspicuous increase in the median UIE value, goitre prevalence was still high in older adult populations mostly living in a severe I-deficient environment before beginning I supplementation^(^ ^20^ ^)^. This implies that the time lag to normalise thyroid size requires more than a decade in an area of chronic I deficiency. Interestingly, the present study found that the prevalence of goitre was significantly lower compared with that reported in 2007 in the adult population living in the same rural area. Hence, this confirms the benefits of sustained iodoprophylaxis in abating the frequency of I-deficiency disorders. A potential side-effect of I supplementation is the induction of autoimmune thyroiditis^(^ ^25^ ^)^. Indeed, studies in both humans and animals have demonstrated that I administration may enhance autoimmune thyroiditis through several mechanisms^(^ ^26^ ^,^ ^27^ ^)^. In particular, it has been reported that I (i) has a toxic effect on thyroid cells^(^ ^28^ ^)^, (ii) directly stimulates immune and immunity-related cells^(^ ^29^ ^)^ and (iii) increases the immunogenicity of TgAb, thereby precipitating an autoimmune process at both T- and B-cell levels^(^ ^30^ ^)^. Recently, it has been demonstrated that by unmasking a cryptic TgAb epitope, I contributes to the induction of thyroid autoimmunity in humans^(^ ^17^ ^)^.

In this study, we have observed a significant increase only for TgAb and not for TPOAb in subjects living in a long-standing I-sufficient area compared with subjects with mild/moderate I intake. These results are in agreement with data reported in the literature, demonstrating an increased incidence of thyroid antibodies after the initiation of an effective iodisation programme^(^ ^11^ ^)^. A direct pathogenic role for TgAb in autoimmune thyroid disease has been proposed by restricted epitope recognition patterns and experimental autoimmune thyroiditis produced by passive transfer of TgAb^(^ ^31^ ^)^. It has also suggested that the positivity of TgAb may be an epiphenomenon with no pathogenic significance^(^ ^31^ ^)^, whereas TPOAb are typical of a more advanced thyroid autoimmune involvement^(^ ^32^ ^)^. However, the possibility that a high prevalence of TgAb may represent a marker of future autoimmune thyroiditis cannot be excluded. In our study, we did not find any significant differences between the two investigated areas when analysing the relationship between I intake and frequency of the HT-US. Nevertheless, we observed a significant increase in serum TgAb in HT-US subjects in the subgroup of individuals from the urban area, whereas no significant changes in thyroid antibodies positivity were evident in subjects with a non-HT-US pattern.

It is important to underline that hypoechogenicity is often the only finding in the initial phases of autoimmune thyroiditis, and it may be present even before detecting serum antithyroid antibodies^(^ ^33^ ^)^. Indeed, thyroid hypoechogenicity has been shown to be more sensitive in predicting the development of hypothyroidism than the presence of antithyroid antibodies^(^ ^34^ ^)^. Our data suggest that the hypoechoic thyroid US pattern did not indicate alteration in thyroid function as no significant differences were found for TgAb positivity in subjects with high TSH levels in the urban area compared with those in the rural area.

In conclusion, this cross-sectional study highlighted a decrease in goitre prevalence and an increase in serum TgAb in the adult population living in an area with long-standing I intakes compared with subjects from a rural area with mild I deficiency. We did not observe any change in the pattern of thyroid diseases between the two populations investigated. However, new studies will be necessary to monitor the impact of I prophylaxis as an important part of preventive health care.
